# Multiwavelength Photoacoustic Doppler Flowmetry of Living Microalgae Cells

**DOI:** 10.3390/bios14080397

**Published:** 2024-08-16

**Authors:** Tayyab Farooq, Xiuru Wu, Sheng Yan, Hui Fang

**Affiliations:** 1 Nanophotonic Research Center, Shenzhen Key Laboratory of Micro-Scale Optical Information Technology, Institute of Microscale Optoelectronics, Shenzhen 518060, China; tayyabfarooq2018@email.szu.edu.cn (T.F.); 2100493005@email.szu.edu.cn (X.W.); 2 College of Physics and Optoelectronic Engineering, Shenzhen University, Shenzhen 518060, China; 3 Institute for Advanced Study, Shenzhen University, Shenzhen 518060, China

**Keywords:** photoacoustic Doppler (PAD), Doppler flowmetry, supercontinuum laser, microalgae absorption spectroscopy, microalgae flow measurement

## Abstract

Photoacoustics can provide a direct measurement of light absorption by microalgae depending on the photosynthesis pigment within them. In this study, we have performed photoacoustic flowmetry on living microalgae cells to measure their flow characteristics, which include flow speed, flow angle, flow direction, and, more importantly, the photoacoustic absorption spectrum, all by observing the photoacoustic Doppler power spectra during their flowing state. A supercontinuum pulsed laser with a high repetition frequency is used as the light source: through intensity modulation at a specified frequency, it can provide wavelength-selectable excitation of a photoacoustic signal centered around this frequency. Our approach can be useful to simultaneously measure the flow characteristics of microalgae and easily discriminate their different species with high accuracy in both static and dynamic states, thus facilitating the study of their cultivation and their role in our ecosystem.

## 1. Introduction

Photoacoustics is gaining much interest in imaging technique development [1] because of the involvement of acoustics, making it suffer less scattering [1] and achieve deeper penetration with higher image resolution over other optical spectroscopy techniques [2]. Photoacoustics has been implemented in many versatile applications, such as the characterization of photoacoustic and fluorescence properties of living cells [3], environmental monitoring [4], and flow cytometry [5,6,7,8,9]. Although research on photoacoustic flow cytometry of human blood has been well established [10,11], very limited data exist on such flowmetry of living botanical cells such as microalgae [12].

In this research, we investigated photoacoustic Doppler flowmetry (PADF) using living microalgae cells [13], which exist very commonly in nature and play an essential role in our aquatic ecosystem. Microalgae exist in both fresh and marine waters and are full of proteins, lipids, and essential amino acids. They serve as a food source for marine organisms and as raw materials for human health products [14]. They convert nitrogen and phosphorus from the environment into biomass using light, CO_2_, and water [15]. Their absorption spectrum [16] depends upon the type and concentration of photosynthesis pigments [17] inside them, such as chlorophyll, fucoxanthin, phycoerythrin, and phycocyanin [18]. These pigments absorb different wavelengths of light for photosynthesis, which allows them to thrive in various aquatic environments. They are considered very suitable for investigation as basic living microbodies for biomedical scientific research. Several other spectroscopic techniques to study microalgae exist [19], including ultraviolet–visible spectroscopy [20], fluorescence spectroscopy [21], Raman spectroscopy [22], Fourier-transform infrared (FTIR) spectroscopy [23], and nuclear magnetic resonance (NMR) spectroscopy [24]. Ultraviolet–visible spectroscopy is limited to surface-level analysis; fluorescence spectroscopy requires labels and can suffer from quenching problems; Raman spectroscopy, though detailed, struggles with fluorescence interference and high costs; FTIR spectroscopy faces water interference and special sample preparation requirements; and NMR spectroscopy is comprehensive but complex, expensive, and requires large sample sizes. In contrast, photoacoustic spectroscopy can offer high sensitivity, depth profiling, non-destructive label-free spectral information, low cost, and real-time monitoring capabilities, making it a robust and versatile alternative for the analysis of microalgae absorption spectra [25].

This study builds upon the foundational research on PADF, where the speed of flowing particles having parabolic flow distribution in a cylindrical tube or vessel is measured from their Doppler shift of frequencies [26,27,28,29,30,31,32]. In recent years, the acoustic properties of suspended algae diatom based on photoacoustic effect have been studied [3], where the polarized light scattering, fluorescence, and light-induced acoustic signals of individual microalgae have been measured by using a 445 nm laser [3]. The use of a 445 nm laser ensures the maximum absorption from intercellular pigments of most microalgae. Photoacoustic lifetime imaging has also recently been performed using a 532 nm laser source for algae diatoms [33]. Other interesting advancements include the implementation of photoacoustic on-chip flow cytometry of living microalgae cells [34]. Still, currently, there are only a few reports about the flowmetric characterization of living microalgae cells using photoacoustics. Furthermore, based on our knowledge, performing PADF on microalgae based on the amplitude-modulated supercontinuum laser source has not been reported.

We experimented on different microalgae species [35,36] and observed their characteristics, such as flow speed, flow direction, flow angle, and, most importantly, their photoacoustic wavelength-dependent absorption spectrum in both stationary and moving states. In a stationary position, the photoacoustic signal in the time domain is directly proportional to the optical absorption spectrum of the microalgae [3,37]. On the other hand, the absorption spectrum in the flow state can be extracted from the Doppler-shifted power spectra [27,38,39]. In this way, from flow state measurements, we obtain not only the photoacoustic absorption spectral information from the Doppler signal but also other important parameters, such as flow speed, flow direction, and flow angle simultaneously [40,41].

We use a supercontinuum laser source [42,43,44,45] for the excitation, which provides a narrow bandwidth, high spatial coherence, and collimated light with selectable wavelengths in the visible region. It also provides a high optical output power and maintains a perfect Gaussian beam. Usually, supercontinuum sources with ns pulses have been used in previous photoacoustic studies [46,47]; however, we have developed a method using a picosecond pulse source with a high repetition frequency of 80 MHz and external intensity modulation around 2.25 MHz [48].

Our experimental results reveal that the PAD shift effect enables the discrimination of the absorption features of flowing microalgae with high sensitivity and accuracy. Notably, we observed distinct absorption spectra of different microalgae species corresponding to light-absorbing pigments such as chlorophyll and carotenoids [18,49]. In general, our approach could identify different microalgae species from their photoacoustic absorption spectrum.

## 2. Methods

### 2.1. Sample Preparation

For the preparation of microalgae samples to perform PADF measurements, precultured samples of different microalgae species were obtained (Binzhou Fengyuan Biotechnology Co., Ltd., Binzhou, China). The samples were transferred into a flask with the addition of F/2 growth medium and kept under visible light and in a controlled temperature environment for 72 h to allow the suspended microalgae cells to settle at the bottom. The supernatant was carefully removed from the top, and the sample was transferred to a test tube for centrifugation [50]. The samples were then centrifuged using a desktop high-speed centrifuge (TG16G, KaidaLab, Hunan Kaida Scientific Instrument Co., Ltd., Changsha, China) at 3000 rpm for 5 min, resulting in the living cells settling at the bottom of the centrifuge tube and forming a pellet with increased cell density [51]. It is essential not to centrifuge at higher speeds or for long time periods to ensure the live condition of the samples. At this stage, the water from the top of the test tube was removed, and the concentrated sample at the bottom of the test tube was taken out using a 5 cc syringe. A glass syringe was used, being preferred over a plastic one due to its inertness, sterility, durability, and reusability. This syringe was then mounted on a computer-controlled microfluidic pump (Pump 11 Elite, P/N: 70-4504, Harvard Apparatus, Holliston, MA, USA), which offers enhanced flow performance with a smooth flow rate ranging from 1.28 pl/min to 88.28 mL/min.

A series of experiments were carried out to observe the photoacoustic effect on different microalgae species such as Green Puff (*Chrysophyta*), Golden Brown (*Chrysophyta*), Triangular (*Phaeodactylum tricornutum*), Diatom (*phytoplankton*), and Green Algae (*Chlorella*) [16]. Each species shows different photoacoustic absorption depending on the photosynthesis pigment inside them. Upon analysis, it was found that the results obtained from green microalgae (*Chlorella*) and golden-brown (*Chrysophyta*) microalgae exhibited the most distinct differences in their absorption peaks, prompting us to select these two microalgae for our further multiwavelength experiment.

Furthermore, because the photoacoustic absorption of *Chlorella* is stronger and its stability at room temperature and in ambient light is far better than that of *Chrysophyta*, it is selected for the demonstration of measuring other flow parameters, such as flow speed, flow direction, and flow angle. These flow parameters are independent of absorption discrimination.

### 2.2. Experimental Setup

**Figure 1:**
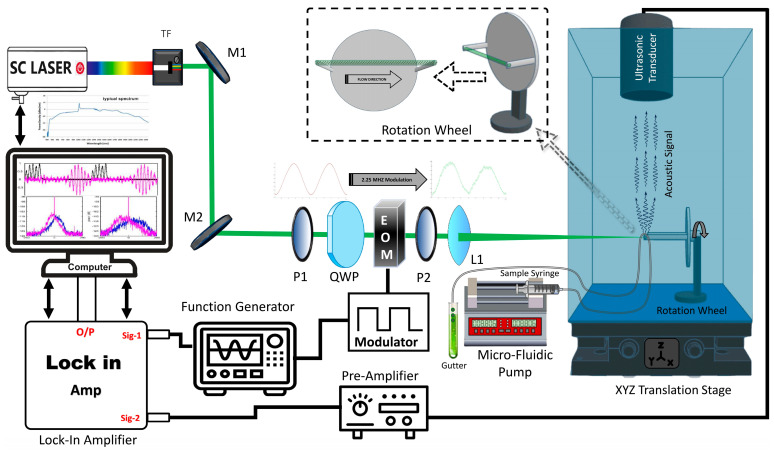
Schematic Diagram of the Experiment. (M1, M2) Mirrors, (QWP) Quarter-Wave Plate, (TF) Tunable Filter, (EOM) Electro-Optical Modulator, (P1, P2) Glan Prisms, and (L1) Convex Lens.

A schematic representation of the experimental setup is depicted in Figure 1. The core device is the supercontinuum fiber laser (Fianium Whitelase SuperK Evo, P/N: FS472-155-010, NKT Photonics, Birkerød, Denmark), which serves as the light source for sample excitation. The laser source is equipped with a tunable filter monochromator (Superk Varia, P/N:A301-100-000, NKT Photonics, Birkerød, Denmark), which transforms the broadband supercontinuum laser into powerful narrow-band lasers with the center wavelength in a wide tunable range of 440 nm (from 400 nm to 840 nm) and with variable bandwidth. The monochromator exhibits a high transmission rate with a maximum bandwidth FWHM of 100 nm and absolute wavelength accuracy ± 5 nm. The laser source is capable of generating continuous white light output made up of high repetition rate (from 20 MHz to 80 MHz) pulses of 250 ps duration with each pulse energy of 92 nJ. It has the highest available output power of up to 7.2 W across the whole emission spectrum (visible spectrum power: 1.5 W), spanning from 400 nm to 2400 nm.

Initially, the laser beam generated from the supercontinuum source is passed through a set of reflecting mirrors (M1, M2) to align parallel with an electro-optical modulated optical path, which consists of Glan prisms (P1) that separate the ordinary and extraordinary components based on their polarization states, a quarter-wave plate (QWP), and a non-resonant electro-optic modulator (EOM, Dv-R3532, S/N:JB815, QUBIG GmbH, München, Germany) before it is transmitted through a second Glan prism (P2). Two clock-synchronized sinusoidal waves with a slight frequency difference of around 2.25 MHz were generated from a function waveform generator (Keysight 33600A Series, Keysight, Santa Rosa, CA, USA), where one of them was attached to the EOM to achieve the desired intensity modulation, and the other was fed into the lock-in amplifier (Zurich Instruments, HF2LI, Zurich Instruments AG, Zürich, Switzerland) reference channel input #1, which works as the reference signal input.

The modulated laser beam was then focused onto a sample of microalgae cells flowing within a transparent silicon tube (Tygon Microbore Tubing, MICROMIX-S-54-HL, Total Plastics International, Wyoming, MI, USA). This action excites the flowing living microalgae cells, producing a photoacoustic signal, which was then detected by an ultrasonic transducer (2.25 MHz/1.00 inch/F = 2.0 inch, I8-0216-P, Olympus Panametrics NDT 2, Olympus IMS, Tokyo, Japan) that was precisely aligned perpendicular to the direction of the flowing microalgae cells to capture their acoustic signal at its maximum value.

Because a narrow-band ultrasonic detector with a center frequency of 2.25 MHz was used in our experiment, we set the EOM modulation frequency identical to 2.25 MHz (to easily differentiate the flow direction, we set the reference frequency slightly higher than 2.25 MHz by 5 Hz). The ultrasonic transducer, which was situated at the top of the water beaker, detects the photoacoustic signals generated from the flowing microalgae samples. As the photoacoustic signal generated by living microbodies was very weak, a signal amplifier (Olympus 5660C Pre-amplifier, Olympus Corporation, Center Valley, PA, USA) was connected to the ultrasonic transducer to boost the input signal strength. The pre-amplifier provides a voltage gain of 60 dB, significantly increasing the system’s sensitivity and allowing for accurate measurements of the photoacoustic signals from the flowing cells. The amplified signal was boosted to the millivolts scale and fed into a lock-in amplifier input #2. The lock-in amplifier also required a reference signal at input #1, which was achieved from the function waveform generator. The lock-in amplifier extracts signal information by de-modulating the received signal to isolate the specific frequency component of interest from the noise [52,53].

The living microalgae cells, which were suspended in water, flow inside a 0.5 mm diameter (1.5 mm outer diameter, 0.5 mm inner diameter) transparent silicon tube. One end of this tube was connected to a microflow pump via a glass syringe (which contains samples), and the other end was attached to a gutter that stores the processed samples for reuse purposes again. The microflow pump was controlled by a computer that was capable of controlling the flow rate to as slow as 0.01 mm/s. The sample transport silicon tube has an outer diameter of 1.5 mm and an inner diameter of 0.5 mm. The syringe utilized had a capacity of 5cc with an inner diameter of 12.55 mm. The microfluid pump controls flow speed by counting revolution steps per minute, so microalgae flow mean velocity was easily and precisely calculated from the above parameters.

To facilitate directional control, the flow pipe was mounted on a spin wheel-shaped disc. This rotating disc can rotate the flow pipe from 0° to 360° to observe the behavior of the flow at different angles [54]. The entire assembly, including the sample, flow pipe, spin wheel disc, and ultrasonic transducer, was placed inside a cubic transparent acrylic beaker filled with distilled water. The filled water acts as the conduction medium for the acoustic signals that were generated from the flowing microalgae samples and detected by an ultrasonic transducer. For XYZ translational control, the water beaker was mounted on an XYZ linear stage (P/N: 16FAS-3L, SK-Advanced, Kadima, Israel), which allows precise adjustment of the sample position in relation to the focused laser spot.

### 2.3. Signal Processing

The power spectral density (PSD) calculation is crucial in the context of PADF measurements because it provides a detailed representation of how the power of the photoacoustic signal is distributed across different frequencies. The PSD is calculated as the square of the magnitude of the fast Fourier transform (FFT) of the signal, which includes both the real (X(f)) and imaginary (Y(f)) parts and then normalized by the effective noise bandwidth (ENBW). The ENBW depends on the windowing function applied during the FFT. The hamming window is applied during the FFT to filter out the noise and unwanted signal from captured data, which affects the ENBW and, consequently, the PSD calculation [55]. Denoting X(f) and Y(f) as the real and imaginary parts of the FFT of the signal, respectively, the PSD can be calculated as ${\left|FFT(X+iY)\right|}^{2}$ and is typically expressed in units of V^2^/Hz.

(1) $$PSD\left(f\right)=\left(\frac{1}{ENBW}\right)\cdot {\left|FFT\left(X+iY\right)\right|}^{2}$$

These FFT results were then processed, enabling us to extract vital signal information parameters, such as area under curves, absorption peaks, and Doppler shift. This comprehensive approach allowed us to analyze the photoacoustic flowmetry of the microalgae cells with high precision and reliability.

We applied the Gaussian fitting on each PSD curve, which was calculated using the Levenberg–Marquardt algorithm below.

(2) $$y=y_{o}+a\times e^{-\frac{{\left(x-x_{c}\right)}^{2}}{2w^{2}}}$$

where $y_{o}$ is the background noise, $x_{c}$ is the center of the axis, a denotes the peak amplitude, and w represents the width of Gaussian fit. The area A under the peak was calculated by a × 2 × w ×$\;\sqrt{\bar{\pi }∕2}$, and the FWHM was calculated by $2\sqrt{\ln\left(4\right)}\times w$.

## 3. Results

### 3.1. Absorption Spectra of Algae Samples in the Stationary State

**Figure 2:**
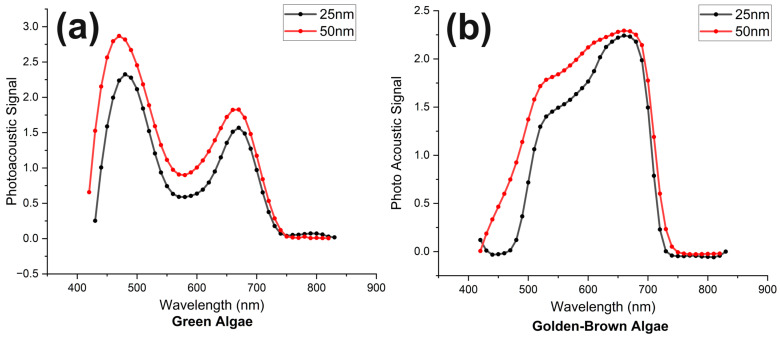
(**a**) Photoacoustic spectra of green algae (*Chlorella*) (bandwidths: black = 25 nm, red = 50 nm). (**b**) Photoacoustic spectra of golden-brown algae (bandwidths: black = 25 nm, red = 50 nm).

In order to obtain the photoacoustic absorption spectrum, the sample from the cultural flask was transferred into a glass syringe and then loaded onto a microfluid flow pump. The pump pushed the sample from the syringe to the silicon tube until it reached the central part of the rotation wheel, where it was exposed to an incident laser beam. The flow speed for the photoacoustic absorption spectrum of the microalgae cells was set to zero during this measurement. Subsequently, the supercontinuum laser beam was focused on the samples, exciting the living microalgae cells, resulting in the production of acoustic signals, which were then detected by the ultrasonic transducer heading on the top perpendicular to the flow tube. The laser was tuned on different wavelengths in the visible region from 425 nm to 825 nm with a tuning interval of 10 nm to observe the photoacoustic absorption of microalgae at each wavelength. The ultrasonic transducer converts the acquired acoustic signal into a voltage scale, which is directly proportional to the light absorbed by algae cells. This voltage signal after amplification was acquired in the time domain using a lock-in amplifier in real time. We recorded the voltage amplitude at each wavelength for an average of 10 s to achieve stable values. After subtracting the unwanted background noise from the obtained results and normalizing it with laser optical power at each corresponding wavelength (with the power meter), the photoacoustic absorption spectrums obtained for both types of algae are shown in Figure 2.

From Figure 2, it is observable that green microalgae (*Chlorella*) exhibited two distinct peaks at 480 nm and 680 nm, respectively, while golden-brown (*Chrysophyta*) microalgae showed a single peak in a very broad window of visible wavelength ranging from 500 nm to 700 nm. The presence of chlorophyll a and chlorophyll b inside green algae cells accounts for the two peaks around 680 nm and 480 nm. On the other hand, the broad absorption window shown for golden-brown algae starting from 480 nm to 700 nm is because, besides chlorophyll a and chlorophyll c (responsible for 680 nm and 480 nm), there is an additional light-absorbing pigment in them called fucoxanthin (responsible for absorption at 580–600 nm) [56].

To use more laser optical power to achieve the desired photoacoustic signal strength, we used two relatively broader bandwidth spans, i.e., 25 nm and 50 nm. The light with 25 nm bandwidth provides spectrally dense output with high wavelength accuracy but with limited total spectral power, while the light with 50 nm bandwidth, on the other hand, offers a broader spectral span, which increases total optical power. The absorption spectra of both bandwidths showed similar photoacoustic absorption behavior with each other, which validates the accuracy of the measured results.

### 3.2. Flow Speed Measurement at Horizontal Flow Direction

In order to achieve the maximum photoacoustic signal, we set the flow direction horizontal (thus, with the flow direction being perpendicular to the ultrasonic transducer axis) to capture the photoacoustic signal at its maximum. For flow speed measurement in this case, we were mainly focused on the width of the PSD curve, and the peak Doppler shift should be very close to zero. Because the sample flow is carried out in a thin silicon tube, the flow profile inside the tube exhibits parabolic velocity distribution, resulting in a higher fluid velocity at its center compared to the velocities near the tube walls (approximately zero). For precise observation of the PAD shift of microalgae cells, these cells need to be distributed uniformly within the water as they pass through a thin silicon tube. Uniform distribution avoids the problem of aggregation, which simplifies the analysis and interpretation of data. This consistency helps eliminate variables that could skew the results, such as turbulence or variations in flow that could affect the pattern of the Doppler shift.

The *Chlorella* samples were measured at horizontal flow direction at different flow speeds to observe its PAD shift power spectrum. A wavelength of 480 nm has been used to ensure maximum photoacoustic absorption. By evaluating all results at different speeds, the graph for five observable flow speed power spectra is shown below.

**Figure 3:**
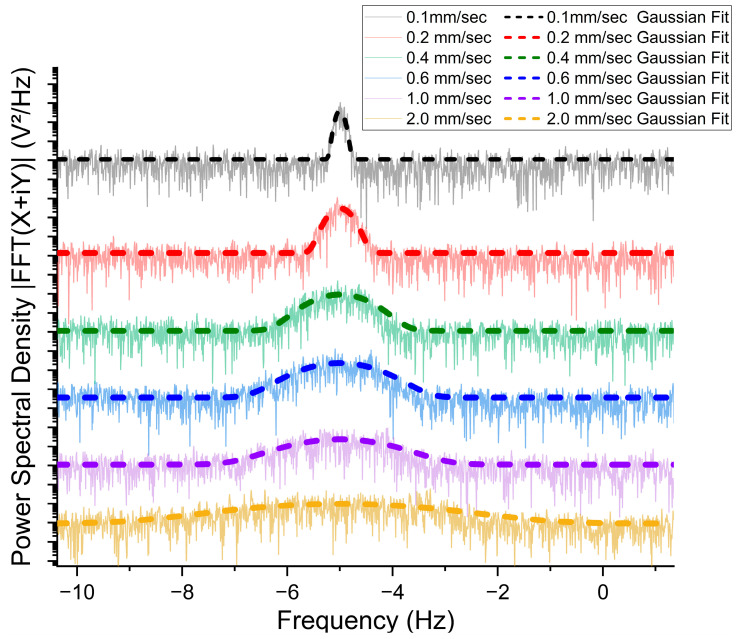
Experimental PAD spectra results for horizontal flow direction and flow speed at 5 different speeds with their smoothed Gaussian approximation collectively.

Because the lock-in reference frequency was set at 5 Hz higher than the laser modulation frequency, the power spectra were distributed around −5 Hz (as shown in Figure 3). For the curves in Figure 3, the background noise was removed, and unwanted high peaks at −5 Hz and 0 Hz (which corresponds to zero speed sample and the free space transportation of the electromagnetic waves) were trimmed off to apply the Gaussian fitting and observe the Doppler shift more clearly. The Hamming window was employed in the Fourier-transformed signal. Furthermore, Gaussian fitting was then applied to each FFT signal to clearly observe the PSD broadening effect. It is obvious in Figure 3 that increasing flow speed results in broadening the width of the PSD curve while keeping the enclosing area constant (maintaining the total power) [32].

In Table 1, the amplitude and width measurements for different flow speeds and the extracted parameters from the Gaussian fitting for each average flow speed are listed and compared. It is obvious that the PSD enclosing area remains almost constant for all speed variations, as when the width w increases, the amplitude a decreases and vice versa. It reflects the fact that the total photoacoustic energy remains conserved for all different speeds. For a typical example, Table 1 shows accurately that the Doppler broadening width w of 2.0 mm/s is about ten times that of 0.2 mm/s, which correlates to ten times the speed.

### 3.3. Flow Direction Measurement

**Figure 4:**
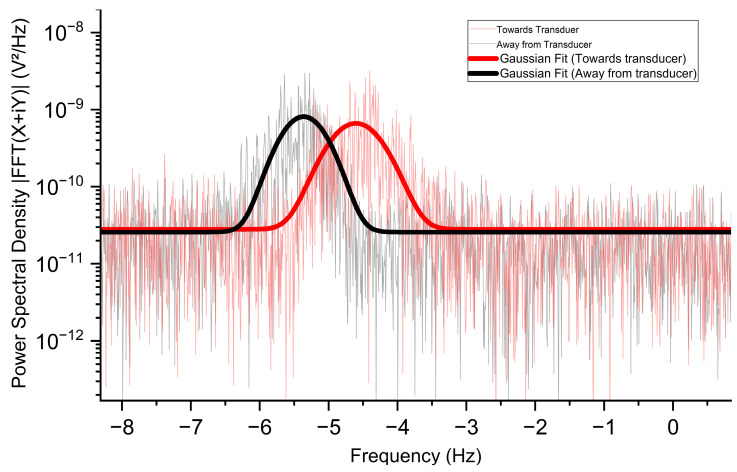
Experimental PAD spectra results for flow direction measurements along with their smoothed Gaussian approximation signal at 0.5 mm/s flow speed and 70° flow angle. Red: flow direction toward the ultrasonic transducer. Black: flow direction away from the ultrasonic transducer.

The direction of flow is interesting information [57] and could not be accessed in the horizontal flow direction measurement. We rotated the silicon tube to 70° (the horizontal direction is at 90°) and set the flow speed as 0.5 mm/s to achieve a visible Doppler shift. The 480 nm wavelength was also used to ensure maximum photoacoustic absorption by the microalgae cells. As mentioned above, we set a gap of −5 Hz between the modulation frequency and the lock-in reference frequency; this allowed us to detect positive or negative Doppler shifts around −5 Hz. The flow direction measured in two opposite directions at the same speed and the same angle is shown in Figure 4.

In the curves shown in Figure 4, the peaks at −5 Hz and 0 Hz have been trimmed off for application of the Gaussian fitting to clearly observe the behavior of shift direction. The red curve is shifted towards the right side of -5 Hz and shows a positive Doppler shift, which agrees with that the flow direction of streaming microalgae samples was flowing towards the ultrasonic transducer. On the other hand, the black curve showed a negative Doppler shift towards the left side of -5 Hz, which agrees with the fact that the flow was flowing away from the ultrasonic transducer. Because the flow speed, flow angle, and excitation wavelength for both curves remained the same, no other differences were observed in the Doppler shift spectrum other than the direction of frequency shift. Actually, the flow direction can be determined easily for different settings of flow speed, wavelength, and flow angle (except the horizontal flow) just by observing the direction of the Doppler shift relative to the zero-frequency shift (−5 Hz in this study).

### 3.4. Flow Angle Measurement

To determine how the PAD power spectra were affected by various flow angles [54,57], a series of experiments were conducted at different flow angles ranging from θ = 0° to 50°, 60°, 70°, 80°, and 90° with a constant flow speed of 0.5 mm/s. The flow angle (θ) is the angle between the flow direction of the algae cells within the tube and the ultrasonic transducer detection axis. The mean PAD shift ∆f is theoretically calculated using the following formula:

(3) $$∆f=2.25\;MHz\times \frac{0.5\;mm/s}{1.5\times 10^{6}\;mm/s}\times cos\theta$$

**Figure 5:**
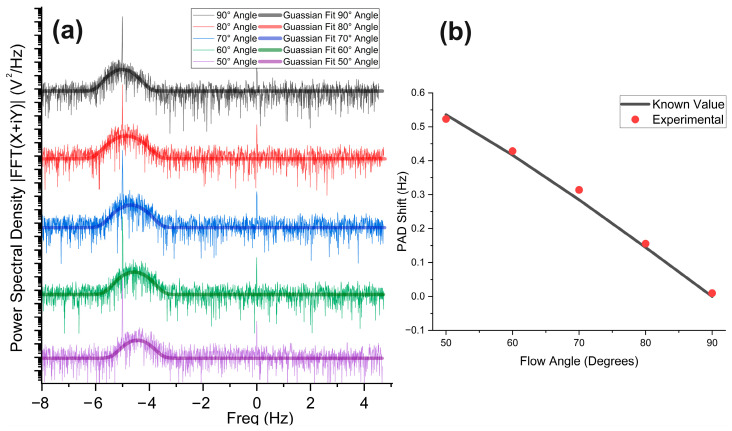
(**a**) Experimental PAD spectra results for flow angle measurements at different angles with their smoothed Gaussian approximation signal. (**b**) Mean PAD shift calculated from power spectra (red dots) compared with theoretical values (black curve) at different angles.

In the above equation, 2.25 MHz represents the laser intensity modulation frequency, 0.5 mm/s is the average flow speed, 1.5 × 10^6^ mm/s is the speed of sound in water, and cosθ represents the cosine of the flow angle. The results are plotted in Figure 5 below for the mean PAD shift.

The PAD shift spectra in Figure 5 show a significant effect of changing the cosine angle. At θ = 90°, the power spectrum is symmetrical around the zero shift (−5 Hz), meaning there is no net Doppler shift. Further measurements with θ = 80°, 70°, 60°, and 50° show an asymmetrical effect around -5Hz in the PAD shift spectrum, resulting in more and more significant positive Doppler shifts. Subsequently, the Gaussian fitting is applied in Figure 5a and shown in thick lines, and the result of calculated and measured PAD shifts versus the flow angle are plotted and compared in Figure 5b.

### 3.5. Photoacoustic Doppler Shift Absorption Spectrum

The last part of this research involved measuring and analyzing PA wavelength absorption from its PAD shift spectrum. For comparison, the PA absorption measured in Figure 2 was carried out by measuring the photoacoustic absorption intensity at the stationary state in the time domain, while here we are calculating the photoacoustic absorption at a flowing state in the frequency domain by observing the behavior of its PAD shift. At the same time, we compared both results to verify our measurement. Here, we set the flow speed at 0.5 mm/s with a flow angle of 70° and flow direction towards the ultrasonic transducer of our microalgae samples.

**Figure 6:**
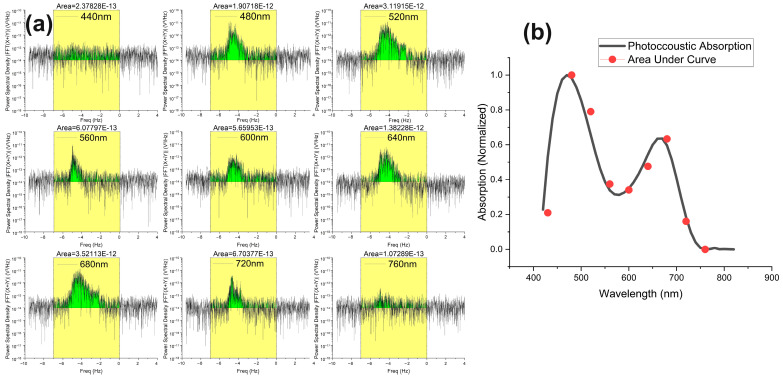
(**a**) Experimental PAD spectra results for green algae at different wavelengths (constant speed, angle, and direction). (**b**) Normalized plot of photoacoustic absorption spectrum in stationary position (black line) and photoacoustic absorption spectrum in flow by estimating area under curve of PAD spectrum (red circles).

In the first case, we took green *Chlorella* and found their PAD absorption spectrum at different wavelengths ranging from 440 nm to 760 nm by the spectral interval of 40 nm and 50 nm bandwidth span. The recorded PSD spectra at each wavelength are shown in Figure 6.

As shown in Figure 6a, we recorded the PAD shift spectra at nine different wavelengths to calculate the area under the curve (AUC). The yellow highlighted block is the part that is selected to be integrated (i.e., −7 Hz to 0 Hz) to find the AUC, and the red line on each graph is the baseline. The green color shades represent the AUC of each PSD spectra. Each graph is marked with the calculated AUC value at the top of every yellow integral window. One thing that needs to be clarified here is that since the PSD is calculated by taking the square of the magnitude of the FFT signal and is measured in V^2^/Hz, the integral AUC of each graph is actually realted to the square of the photoacoustic absorption spectrum (i.e., voltage). Therefore, we further calculated the square root of this area and normalized it with laser optical power to calculate the photoacoustic absorption intensity at each wavelength. Figure 6b shows all of such calculated data points at different wavelengths and plotted in red dots, while the black curve is the photoacoustic absorption curve measured in stational position, which is taken from the 50 nm bandwidth curve of Figure 2a to compare. It is clearly observable that both static and dynamic measurements (black line and red dots) are following each other, thus proving our measurement approach.

**Figure 7:**
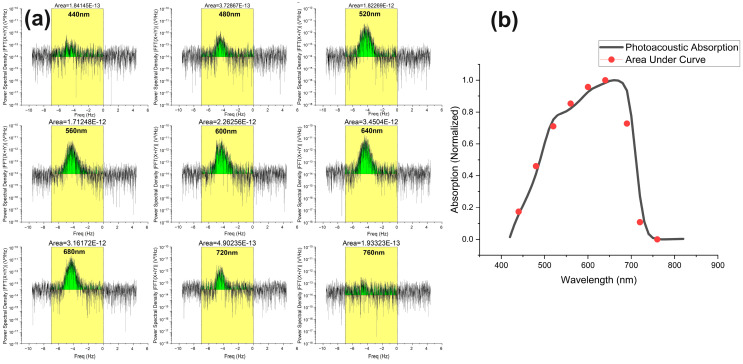
(**a**) Experimental photoacoustic Doppler spectra results for golden-brown algae at different wavelengths (constant speed, angle, and direction). (**b**) Normalized plot of photoacoustic absorption spectrum in stationary position (black line) and photoacoustic absorption spectrum in flow by estimating area under curve of PAD spectrum (red circles).

Similarly, we also performed the above measurement and analysis for the golden-brown microalgae, where the PAD spectra of different wavelengths in the visible light region have been recorded in the same way. Their PSD spectra were integrated within the selective frequency range to find the AUC shown in the shaded green color in Figure 7a. The red circles in Figure 7b represent the data points calculated from Figure 7a, and the black curve is taken out from Figure 2b to compare the trend of photoacoustic absorption in stationary and moving states.

## 4. Discussion

The distinct photoacoustic absorption of two different microalgae species using a supercontinuum laser source has been well-demonstrated here. The photoacoustic absorption of different microalgae species depends upon the concentration and types of photosynthesis pigments inside them. The golden-brown microalgae (*Chrysophyta*) consist of three primary absorption pigments, i.e., chlorophyll a, chlorophyll c, and fucoxanthin. In comparison, green microalgae (*Chlorella*) consist of two primary absorption pigments, chlorophyll a and chlorophyll b. It is imperative to delineate the distinctive absorption peaks of these pigments. Chlorophyll a exhibits an absorption peak at 680 nm, while chlorophyll b and c exhibit nearly the same absorption peak between 445 and 470 nm [58,59]. The absorption spectra of both types of microalgae have been measured in a stationary state and are shown in Figure 2. Distinct and observable differences in absorption peaks have been observed, with two peaks and one broad peak, respectively, in *Chlorella* and *Chrysophyta.* The presence of fucoxanthin in golden-brown algae lets them absorb more light in the wavelength range from 550 nm to 630 nm, and with chlorophyll a and chlorophyll c absorption peaks, collectively, it gives a very broad absorption peak from 475 nm to 680 nm [56,60]. This broad absorption spectrum allows them to survive in different light environments, from surface water with high light intensity to deeper water where light is limited.

For all of the PSD graphs presented here, we have taken 2300 data points to construct the PSD curve. The single measurement takes 145 s, resulting in an effective spectral range of 15.0 Hz, maintaining a spectral resolution of 6.862 mHz. Additionally, the first-order low-pass filter has been applied in the lock-in amplifier to reduce high-frequency noise and interference in the measuring signal. These parameters are optimized for capturing PAD shifts within a flow speed range between 0.1 mm/s and 1 mm/s; selecting a higher frequency resolution adds more temporal delay in the lock-in amplifier signal processing, and selecting a lower frequency resolution may not be enough to visualize PAD shifts clearly. Another thing is that all the PAD power spectra drawn in this research work show the Doppler shift around −5 Hz as the central axis. This is because the lock-in reference frequency was set at 5 Hz higher than the laser modulation frequency, which creates a gap of 5 Hz between both frequencies. This offset limits the maximum flow speed calculation to about 2.0 mm/s because the PAD power spectra show broadening, which is directly proportional to the flow speed. For the 2.0 mm/s flow speed, the FWHM is shown to be 3.68 Hz. For measuring flow speeds greater than 2.0 mm/s, a higher offset (above 5 Hz) may be required. However, we have found that the noise ratio increases at high flow speeds. The signal amplitude decreases with an increase in the spectral broadening width, so the flow speed measurement may not be very accurate for the flow speed higher than 2.0 mm/s. As the average flow speed increases, the height of the PAD power spectrum decreases while the width increases, maintaining the total photoacoustic power.

Flow direction measurement is independent of flow speed and flow angle because flow direction is measured by observing the direction of Doppler shift direction (either towards or away from 0 Hz). For the flow angle measurement shown in Figure 5, cosθ is cosine of the angle that comes from the directions between the axis of the ultrasonic transducer and the flow direction. We have measured the values for flow angles 90°, 80°, 70°, 60° and 50°. The graph in Figure 5b shows the relation between theoretical (black curve) and measured (red dots) values which agree with each other. Compared to our previous work [54], we actually obtain better results for the PAD shift at different flow angles. The major reason is because of the improvement in the rotatable angular mount structure on which sample flow has been made. The angle adjustment is more precise on our new mount. There is no net shift at 90° because the Doppler-shifted frequencies are equally distributed on both sides of zero shift frequency(−5 Hz). Theoretically, cos 90° also makes the mean PAD shift ∆*f* to zero in Equation (3). As we decrease the angle, we clearly observe the net shift increasing from 0 Hz to 0.55 Hz at 50°. Due to the limitation in laser optical power and some restrictions in our rotatable mount structure, the photoacoustic signal either went weak or was blocked by the obstacle pillar on which the silicon flow tube was mounted, resulting in very weak detection of the photoacoustic signal at angles below 50°. Therefore, we have limited our measurements to angles of 50° and above.

Usually, before applying the Gaussian fits, we have trimmed down the excess peaks at both −5 Hz and 0 Hz so that we can apply the Gaussian fit more precisely along with the signal. However, in Figure 5a, to show these peaks to observe the PAD shift clearly, we first eliminate the peaks to apply Gaussian fit, then undo the elimination. The Gaussian fit provides a straightforward method to determine the Doppler shift and allows the observation of the parameters of the PSD signal in terms of its width, amplitude, area, center, and FWHM. These parameters allow us to precisely measure the flow speed, the flow direction, and the flow angles.

Even in the absence of incident laser light, we found that some acoustic signal was still detected by the ultrasonic transducer, which was considered background noise. The major reasons for this background noise signal may come from the combination of electromagnetic interference, electronic component noise, and environmental factors. To overcome this background noise, the EOM, the function generators, and the lock-in amplifier were put far away from each other on different tables to reduce electromagnetic interference and electrostatic discharge. This helped reduce the background noise by ten times. For our photoacoustic absorption measurements in the stationary state, it is obvious that the lesser the background noise, the more accurate it will be.

The laser modulation frequency and the central frequency ultrasonic transducer are directly related to both the frequency shifts and the magnitudes observed in the power spectra. The higher frequencies provide better resolution and higher measurable flow speed but result in greater acoustic signal attenuation. On the other hand, lower frequencies enhance the signal-to-noise ratio, making them more suitable for measuring lower flow speeds. The choice of the frequencies thus depends on the specific requirements of the measurement, such as the desired accuracy, signal strength, and flow speed range.

The results in Figure 6 and Figure 7 demonstrate the major novelty of our work. Currently, we have constructed the absorption spectral graphs in dynamic conditions using a wavelength interval of 40 nm from 415 to 850 nm. The nine power spectra shown in Figure 6a and Figure 7a are analyzed to construct the absorption spectra shown in Figure 6b and Figure 7b. The spectral resolution can be increased by reducing the wavelength interval from 40 nm to 10 nm, which will result in a total of 44 power spectra to construct the absorption spectrum in the dynamic flow condition. However, this will make the measurement very time-consuming. Furthermore, we have found that in our experiment, the ideal setting of the flow speed and flow angle for achieving a sufficient Doppler-shifted signal strength is 0.5 mm/s and 70°. For other flow speeds and angles, the measured spectral accuracy could be affected somewhat because of the decreased signal-to-noise ratio.

There are several benefits to measuring photoacoustic absorption spectra with this new approach. First, the PSD spectrum of PAD signal not only gives information about the signal energy but also provides information about other flow characteristics simultaneously, such as flow direction, flow speed, and flow angle. Another benefit is that the calculated absorption spectrum can avoid the inference of the background noise sitting at the 0 Hz shift because of analyzing the PAD-shifted PSD. A further improvement under consideration in our experimentation is to measure the photoacoustic absorption spectrum using Fourier-transform spectroscopy, which would eliminate the need for wavelength tuning.

## 5. Conclusions

We present a novel approach for measuring the photoacoustic absorption spectrum of flowing microalgae cells from their Doppler-shifted power spectra. The use of supercontinuum sources allows us to observe the photoacoustic behavior of living microalgae cells at different wavelengths. The photoacoustic absorption spectra of two kinds of microalgae were well-differentiated and agreed with the results of the conventional methods such as spectrophotometry. We also measured the flow speed, the flow direction, and the flow angular dependence of living microalgae cells. The flow direction and flow speed measurements are particularly useful in applications where visual observation of flow is difficult, such as the slow flow within translucent biological tissues or microfluidic devices. Besides the microalgae pigment study, the technique demonstrated can be further extended to other biological and environmental studies and may offer a new dimension to photoacoustic spectroscopy applications.

## Figures and Tables

**Table 1:** Amplitude and width measurements for different flow speeds.

Average Flow Speed	a (V^2^/Hz)	$x_{c}$ (Hz)	w (Hz)	A (V^2^)	FWHM (Hz)
0.1 mm/s	1.42985 × 10^−9^	−4.99822	0.06144	2.2015 × 10^−10^	0.14468
0.2 mm/s	6.09522 × 10^−10^	−4.99926	0.16522	2.5237 × 10^−10^	0.389063
0.4 mm/s	2.31676 × 10^−10^	−4.99959	0.41571	2.4135 × 10^−10^	0.978922
0.6 mm/s	1.81575 × 10^−10^	−4.99964	0.557	2.5345 × 10^−10^	1.311635
1.0 mm/s	1.30865 × 10^−10^	−4.99701	0.74828	2.4540 × 10^−10^	1.762065
2.0 mm/s	5.89998 × 10^−11^	−4.99464	1.56694	2.3168 × 10^−10^	3.689862

## Data Availability

The original contributions presented in the study are included in the article, further inquiries can be directed to the corresponding author.
